# Current Hospital Topics

**Published:** 1907-02-02

**Authors:** 


### HOSPITAL ADMINISTRATION.

### CONSTRUCTION AND ECONOMICS.

### CURRENT HOSPITAL TOPICS.

#### Melbourne Dental Hospital.

The inhabitants of Melbourne have generously determined to build a new Dental Hospital and to equip it in all respects for the thorough study of modern dentistry. The cost of the new building is estimated to exceed £10,000, and the plans have been prepared in competition by Messrs. Campbell and Kernot. The buildings will be of three stories, with a lecture theatre for 100 students.

#### Workmen's Contributions: a Record.

Mr. Foreman, one of the treasurers, stated at a recent meeting that the workmen of Sir W. G. Armstrong, Whitworth and Co., Limited, employed at the Elswick Works, had raised in penny subscriptions in the last eighteen and a half years £38,685. Of this sum £27,233 had been given to the Newcastle Infirmary. The balance had been distributed amongst the local special hospitals, surgical aid societies, nurses' associations, convalescent homes, and dispensaries. We believe this to be a record contribution by the employees of a single great establishment to the cause of the sick.

#### Bad Management and Wasted Money.

We have received a type-written circular on behalf of some hospital, the name of which is not even mentioned in the document in question. It is signed by someone whose name, though typed, is not legible, above the words "Organising Secretary." It asks for the insertion of a paragraph which has no meaning, seeing that it relates to some hospital the name of which is not given, although it contains the name of a member of the Royal Family. It is difficult enough for capable men of business to raise money for charities, but any committee which does not supervise the work of the secretariat, and permits money to be wastefully expended upon postage for the circulation of a useless document of the kind referred to, is certainly neglecting its duties. We call attention to the matter because the type-written communication is evidently one of a number which have been sent to the Press, and it indicates very clearly why some appeals receive no response. Formerly, when appeal literature produced money, it was prepared with infinite care, in an attractive form, and made a direct and personal appeal to each individual who was approached. This latest specimen of modern appeal literature is lamentable from every point of view.

#### Taxation of Male Servants at Hospitals.

Instances have occurred from time to time where the Inland Revenue officers have sent in demands for payment of taxes on male servants employed by hospitals, with the object of assessing the charities upon the same basis as private individuals. In many cases the duty of 15s. per annum for each male servant has been paid. It should be noted, however, that the Commissioners of Inland Revenue take a very lenient view of the matter. They maintain, it is true, that strictly speaking they are entitled to the duty, but their wish is to grant an indulgence to charities whenever possible. Recently a zealous provincial officer discovered that the local hospital employed two gardeners, and promptly sent in a demand for 30s. This was resisted by the Hospital Committee, and the matter was eventually brought before the Commissioners, who on consideration declared their willingness to regard the gardeners employed by hospitals as "spade labourers," and that as such they should not be chargeable with duty.

#### The Parsimony of the Many.

Nearly every week we have to call the attention of the inhabitants of some large city to the remissness of the majority in regard to hospital contributions. Cardiff has a very useful hospital which is widely popular and does good work. The hospital bed accommodation is only about one per thousand, which is less than the present needs of the community. This is proved by the circumstance that 570 patients are waiting admission and that there are only 184 hospital beds. The population of Cardiff is 180,000, yet the cost of maintaining and administering these beds and the hospital which contains them, is mainly provided by 450 ladies and gentlemen who alone subscribe annually to the funds of the Cardiff Infirmary. Lord Aberdare and the Committee have therefore a strong case when they appeal for an increased income of £3,000. Such a sum ought to be immediately forthcoming in response to the appeal now being circulated. The truth is, so many men and women have acquired the habit of taking everything from the hospitals for nothing, that they are apt to forget that they ought spontaneously to support these great medical charities in the days of health. Other cities and towns throughout the country are waking up to their duty to the hospitals. Will Cardiff be content to slumber in health, and to take everything when ill themselves, whilst the majority of the citizens give nothing systematically to the sick poor?

### Hospitals in Western Australia.

The Report of the principal medical officer, for the year ending June 30, 1906, just issued, contains particulars of the cost of the upkeep of Government and assisted hospitals in Western Australia. Government hospitals on the coast within settled areas vary as to the cost per patient per day from 10s. 10d. at Guildford to 4s. 6d. at Bunbury. The explanation may be that only thirty-six patients were treated at the former hospital, against 385 at the latter. The north-western hospitals, of which there are six, appear to be expensively conducted. Thus, the actual cost per patient per day at Onslow was £1 13s. 6d., at Derby it was 19s. 8d., and at Wyndham 18s. 9d.; whereas at Carnarvon it only amounted to 5s. 2d. per patient. Of the assisted hospitals, that at Ravensthorpe is the most expensive, for the cost per patient per day, exclusive of buildings, was £1 16s. 11d. At Peak Hill it was £1 3s. 9d., and at Mulwarie it was £1 0s. 1d. At Leonora the cost per patient per day was only 7s. 4d., the lowest out of thirteen hospitals in this class. It is not possible to explain the dif- to slumber, and to take everything when ill themselves, whilst the majority of the citizens give nothing systematically to the sick poor?

### St. John's General Hospital, Newfoundland.

This hospital has laboured under considerable disability for years owing to the pressure upon its beds. After a good deal of discussion and consideration, it has been determined to reconstruct the hospital from plans prepared by Sir Aston Webb. These include an extension of the present male block, north, south, and west. There will then be four wards for men, each containing twenty-two beds, a small observation ward for male patients, and a similar one for women, with a new operation theatre situated in the centre of the new building. The scheme includes the conversion of the oldest wing of the hospital into a nurses' home and administration block. The reconstruction of the wards was commenced in the autumn and has made good progress; but the new nurses' home must be delayed until the new house, which is to constitute the residence of the principal medical officer, has been completed and is ready for occupation. Possibly in view of the extension of the hospital, the medical staff has been increased by the appointment of Dr. Macpherson, who has been placed in charge of the $x$-ray and light department.

